# Cefazolin as an alternative in patients with a history of penicillin or β-lactam antibiotic allergy: A case overview 

**DOI:** 10.5414/ALX02634E

**Published:** 2026-05-29

**Authors:** Burkhard Kreft, Johannes Wohlrab, Cord Sunderkötter

**Affiliations:** Department of Dermatology and Venereology, University Hospital Halle (Saale), Martin Luther University Halle-Wittenberg, Germany

**Keywords:** cefazolin, β-lactam antibiotics, cephalosporin, cross-reactivity, side chain

## Abstract

Many patients with suspected or confirmed allergy to penicillin or β-lactam antibiotics are unnecessarily denied all β-lactam antibiotics (BLA) and instead given a less effective antibiotic with more side effects. However, cefazolin, a first-generation cephalosporin, is a safe and effective treatment option for these patients in most cases. Recent studies show that immunological cross-reactions among BLAs are primarily caused by structural similarities in the side chains and not by the common β-lactam ring. Cefazolin has unique R1 and R2 side chains that largely rule out cross-reactivity with other BLAs. In a case series, 21 patients with a history of alleged or confirmed allergy to BLAs were exposed to cefazolin under real-life conditions, and all but 1 patient (who developed an uncomplicated maculopapular rash) showed no reaction. Therefore, the use of cefazolin is acceptable under clinical supervision in cases of a previous uncomplicated delayed-type reaction. However, in cases of reported or documented clinical reactions to BLA that are indicative of a type I allergy (such as urticaria, angioedema, anaphylaxis), prior to (titrated) administration of cefazolin under appropriate monitoring measures, a diagnostic evaluation in accordance with guidelines, including a negative skin test for cefazolin, is advisable. This procedure avoids the use of less suitable reserve antibiotics and ensures effective treatment without incalculable risk for anaphylaxis.

## Introduction 

In patients with a history of alleged or confirmed allergy to penicillin or other β-lactam antibiotics (BLA), other antibiotics from this substance group are often not administered due to concerns about possible immunological cross-reactions. Instead, an antibiotic with a less specific spectrum of activity and therefore more undesirable side effects for patients, as well as the risk of selecting resistant bacteria, is given. However, according to current knowledge, the extent of cross-reactivity appears to be significantly less significant than previously assumed [1, 2]. We now know that, apart from a few isolated cases, immunological cross-reactions, particularly between penicillins and cephalosporins, are usually caused not by the β-lactam ring structure, but primarily by similarities in the R1 and, to a lesser extent, the R2 side chains [3, 4]. Since cefazolin, a first-generation cephalosporin, has an R1 and R2 side chain structure that differs from those of all other BLA currently approved in Europe and the USA, cross-reactivity with other BLA due to the side chains is not to be expected in most cases [4, 5, 6, 7]. Cefazolin is therefore usually a suitable alternative therapy for patients with documented or suspected penicillin allergy [8]. 

For 2 years, our clinic has been treating patients with a history of non-life-threatening, delayed-type allergy to penicillin or other BLA (except cefazolin) with cefazolin when indicated, under appropriate monitoring precautions and without immediate prior in vivo or in vitro allergy testing. In this case overview, we summarize the courses of treatment under the “real-life conditions” of everyday clinical practice. 

## Case overview 

Between 2022 and 2024, 19 patients with a history or documented allergy to BLA were treated at our clinic with the following indications: erysipelas (n = 15), soft tissue infection of the skin (n = 4), and tonsillopharyngitis (n = 1) who required inpatient intravenous antibiotic therapy due to the urgent need for treatment and the lack of facilities for extended allergological diagnostics. 

In cases where the patient’s medical history indicated previous reactions to a BLA suggestive of a type I allergy (urticaria, angioedema, anaphylaxis), titrated exposure with cefazolin was only performed after a negative skin test. 

Between 2022 and 2024, 2 patients received cefazolin as part of an alternative testing protocol due to a history of BLA allergy. 

All patients denied any indications of a previous severe drug reaction (severe cutaneous adverse reaction (SCAR)), such as the occurrence of blisters, systemic involvement, or a protracted course of the disease. Information about treatment measures taken following the drug reaction was only available in some of the cases, as most patients could no longer remember this. Analysis of the available patient records yielded further information on the therapy in only a few cases. In 13 cases, it was no longer possible to determine whether the suspected BLA leading to the allergic reaction was administered orally or intravenously. 

One patient with suspected delayed-type allergy to penicillin and amoxicillin/clavulanic acid developed a maculopapular delayed-type exanthema within 24 hours of starting cefazolin treatment. None of the remaining 18 patients experienced an immediate or delayed-type allergic reaction. 

The titrated exposure with cefazolin after prior negative skin testing (prick and intradermal testing) carried out as part of an allergological investigation in a patient with a confirmed immediate-type allergy to cefuroxime (history of intraoperative anaphylaxis in temporal connection with intravenous administration of cefuroxime, evidence of type I sensitization in the intradermal test) also proceeded without any reaction. A patient with confirmed delayed-type allergy to penicillin and ampicillin (history of “facial swelling” in temporal association with penicillin intake and “exanthema” after amoxicillin intake, evidence of type IV sensitization to penicillin and amoxicillin in the patch test and to penicillin and ampicillin in the intradermal test) tolerated an alternative exposure with cefazolin (Table 1). All patients received cefazolin in an inpatient setting under appropriate monitoring precautions and with a doctor present in the immediate vicinity. 

## Discussion 

Cefazolin is a first-generation cephalosporin and, due to its excellent safety profile and targeted efficacy against common pathogens such as *S. aureus* or *S. pyogenes*, is used, among other things, for the prophylaxis of postoperative wound infections and for the treatment of skin and soft tissue infections [9]. In the past, cefazolin was often not administered intraoperatively or postoperatively to patients with suspected penicillin allergy in order to avoid possible cross-reactions. Instead, non-BLA antibiotics such as clindamycin, vancomycin or fluoroquinolones were and continue to be used as alternatives, which, for example, not only offer lower safety and efficacy in the context of antibiotic prophylaxis, but also have more side effects and lead to an increased risk of postoperative wound infections in patients labelled as penicillin-allergic [10, 11, 12]. Of particular note is the increased risk of developing complicated infections with *Clostridioides difficile* or vancomycin-resistant enterococci [11, 13]. Previous assumptions about frequent cross-allergenicity between penicillins and cephalosporins were based on presumed production-related contamination of cephalosporins with penicillins until the mid-1980s [14], which, when a history of penicillin allergy is reported, still leads to the mostly unjustified avoidance of all BLA. It is now well known that in the vast majority of cases of allergy to a BLA, sensitization is directed against side chain structures and that cross-reactivity with regard to an antigenic determinant of the β-lactam ring, which would lead to positive allergy test results for all β-lactams, is very rare in individuals with IgE-mediated hypersensitivity and may not be observed at all in T-cell-mediated hypersensitivity [15, 16]. 

Interestingly, cefazolin has a unique combination of R1 and R2 side chains that is only found in ceftezol, an antibiotic marketed exclusively in Asia [17]. Studies [6, 7] show that in cases of confirmed penicillin allergy, the probability of cross-reactivity to cefazolin is low (0.8 – 3%). A meta-analysis of 77 studies found that the frequency of simultaneous penicillin and cefazolin allergy in surgical patients was 0.7%; and only 0.1% in cases of unconfirmed penicillin allergy [7]. 

A cross-sectional study [18] showed a very low prevalence of perioperative anaphylaxis in patients with penicillin allergy who received cefazolin prophylaxis (< 0.1%). Nowadays, it can be assumed that supposed cross-reactions are more likely to be due to co-sensitization than to genuine reactions to an antigenic determinant of the common β-lactam ring structure [6, 18, 19]. 

In 2017, Vancouver General Hospital, in collaboration with pharmacists, anesthetists, and surgeons, initiated a program whereby all patients with a documented penicillin allergy, including those with previous anaphylactic reactions, receive cefazolin as part of an indicated prophylaxis for postoperative wound infections. The only exceptions to this were patients who had suffered a SCAR such as Stevens-Johnson syndrome (SJS), toxic epidermal necrolysis (TEN) or DRESS. The evaluation showed that even in the presence of IgE-mediated penicillin allergy, cefazolin can be used for preoperative antibiotic prophylaxis [20, 21]. However, it is important to note that cefazolin itself can trigger anaphylactic reactions. In some countries, such as Spain and the USA, cefazolin is even one of the more common triggers of perioperative anaphylaxis [22, 23, 24]. However, this is usually a case of de novo sensitization to cefazolin after previous administration and not immunological cross-reactions due to a side chain structure similarity with other BLA. Therefore, allergic reactions to cefazolin are to be expected, regardless of whether there is a history of previous reactions to penicillin or other BLA. 

However, even in patients with less clearly defined symptoms following the intake of BLA – such as non-specific symptoms (palpitations, dizziness, headaches) without any further signs of anaphylaxis – a BLA allergy is often suspected, and a corresponding certificate is issued without further investigation. 

Under “real-life conditions” in everyday clinical practice, we have administered cefazolin without prior allergy skin testing to patients with a history of penicillin allergy, the manifestation of which was often described as a delayed-type drug rash, without any severe reactions occurring. Our observations support the use of cefazolin in patients with delayed hypersensitivity reactions to BLA in clinical practice even if the first dose should be administered under observation and with readiness to treat an immediate-type allergic reaction, just to be on the safe side. Many patients can no longer describe the exact reaction, as it often occurred many years ago. In many cases, it is also no longer possible to determine whether symptomatic treatment of the “allergic” reaction that occurred was necessary. As a rule, however, it can be assumed that a patient who has survived anaphylaxis and required appropriate treatment can also remember it. In addition, most patients can indicate whether they have ever been given an antibiotic parenterally and not just orally. If BLA has only been administered orally to date, this essentially rules out primary sensitization to cefazolin, which is available only for parenteral use. 

If there is a history of immediate-type allergy to penicillin, guideline-based diagnostic testing [16] is recommended prior to titrated exposure to cefazolin (starting dose 1/10, followed by a full dose 30 minutes later) under appropriate monitoring precautions, as sensitization to the β-lactam ring may exist, albeit with a low probability, and an immunological cross-reaction with cefazolin is therefore possible [16]. It seems sensible, particularly in cases of anamnestic BLA allergy with a risk of a severe immediate reaction, to perform a skin test before administering cefazolin [25]. 

### Limitations 

It should be noted that our observations are based on a small number of cases and that it is often unclear whether an allergic reaction or rather an infection-related exanthema was present in the clinical picture of a maculopapular exanthema or infection-associated urticaria. However, this question cannot always be answered with certainty in everyday clinical practice. 

### Conclusion for practice 

In view of the considerable disadvantages and risks of second-line non-BLA therapy in patients labelled as penicillin-allergic in situations where cefazolin would actually be the treatment of choice, we believe that optimized practice guidelines are needed. A position paper by various allergology societies was recently published, which addresses the recommendations for the procedure in cases of reported allergy to penicillin/BLA [25]. When weighing up the benefits of guideline-compliant antibiotic therapy with cefazolin, where indicated (prophylaxis of postoperative wound infections, treatment of uncomplicated soft tissue infections of the skin), against the extremely low potential risk of an allergic reaction to this active ingredient in patients labelled as penicillin-allergic, the risk of allergic reactions when administering cefazolin appears to be low especially in patients with a history of uncomplicated maculopapular exanthema (or other, rather unspecific symptoms such as palpitations or headaches), even without prior allergy testing. In our study, the benefits of using a suitable antibiotic clearly outweighed the risks of uncomplicated drug rash in patients with uncomplicated maculopapular rash or unknown reactions in their medical history. However, in patients with a history of IgE-mediated (anaphylactic) reactions to penicillin or another BLA, it is advisable to perform diagnosis in accordance with guidelines for BLA, including a negative skin test to cefazolin before administering cefazolin (titrated) under appropriate monitoring measures. 

This approach should also be taken into account in the S2k guideline on the treatment of bacterial skin and soft tissue infections, which is currently being updated [9, 26]. 

Optimized practice guidelines are required to enable the more targeted and safer use of cefazolin in cases of known penicillin allergy. It would be desirable if corresponding recommendations were also taken into account in future guideline recommendations. 

## Authors’ contributions 

Burkhard Kreft (BK) and Cord Sunderkötter (CS) contributed in acquisition and interpretation of data for the work. BK drafted the manuscript. Johannes Wohlrab (JW) and CS contributed to the content. All authors have given final approval for publication of the manuscript. 

## Funding 

No external funding was received for this work. 

## Conflict of interest 

The authors have no conflict of interest to declare. 

**Table 1.** Patients with history of β-lactam antibiotic allergy and exposure to cefazolin.

**No.**	**Allergy**	**Application**	**Documentation**	**Reaction**	**Treatment**	**When**	**Allergological diagnostics**	**Indication**	**Exposure to cefazoline**
1	Penicillin	?	Allergy passport/card	Exanthema	?	?	Not done	Erysipelas	Tolerated
2	Penicillin	?	Medical history	?	?	?	Not done	Erysipelas	Tolerated
3	Penicillin	?	Medical history	?	?	Childhood	Not done	Erysipelas	Tolerated
4	Penicillin	?	Allergy passport/card	?	?	?	Not done	Soft tissue infection	Tolerated
5	Penicillin	?	Medical history	?	?	About 15 years ago	Not done	Erysipelas	Tolerated
6	Penicillin Amoxicillin	?	Allergy passport/card	Exanthema	?	1998	Not done	Erysipelas	Tolerated
7	Penicillin	?	Medical history	?	?	?	Not done	Soft tissue infection	Tolerated
8	Amoxicillin	PO	Allergy passport/card	Exanthema	No	2006	Type-IV-sensitization to amoxicillin and ampicillin (skin test)	Erysipelas	Tolerated
9	Amox/Clav	PO	Allergy passport/card	Exanthema	GK, AH	2024	Not done	Tonsillopharyngitis	Tolerated
10	Penicillin	IV	Allergy passport/card	Exanthema	No	2009	Not done	Erysipelas	Tolerated
11	Penicillin	?	Allergy passport/card	?	No	?	Not done	Erysipelas	Tolerated
12	Cefuroxim	IV	Allergy passport/card	Anaphylaxis	GK, AH Adrenalin	2023	Type-I-sensitization to Cefuroxim (skin test)	Alternative exposition	Tolerated
13	Amoxicillin	?	Medical history	Exanthema	?	?	Not done	Soft tissue infection	Tolerated
14	Penicillin	?	Medical history	Exanthema	?	?	Not done	Erysipelas	Tolerated
15	Penicillin Amox/Clav	?	Medical history	Exanthema	?	?	Not done	Erysipelas	Maculopapular exanthema
16	Penicillin	?	Medical history	Exanthema	?	2022	Not done	Erysipelas	Tolerated
17	Penicillin	IV	Medical history	Exanthema	?	2017	Not done	Erysipelas	Tolerated
18	Amoxicillin	PO	Medical history	Exanthema	GK, AH	?	Not done	Erysipelas	Tolerated
19	Penicillin Amoxicillin	PO	Medical history	Angioedema Exanthema	No	About 35 years ago 2021	Type-IV-sensitization to amoxicillin and ampicillin (skin test)	Alternative exposition	Tolerated
20	Amp/Sul	IV	Medical history	?	?	2021	Not done	Soft tissue infection	Tolerated
21	Penicillin	?	Medical history	?	?	?	Not done	Erysipelas	Tolerated

Pip/Taz = piperacillin/tazobactam; Amox/Clav = amoxicillin/clavulanic acid; Amp/Sul = ampicillin/sulbactam; GK = glucocorticoid; AH = antihistamine; i. v. = intravenous; p. o. = per os.
